# Efficient counting of ***k***-mers in DNA sequences using a bloom filter

**DOI:** 10.1186/1471-2105-12-333

**Published:** 2011-08-10

**Authors:** Páll Melsted, Jonathan K Pritchard

**Affiliations:** 1 Department of Human Genetics, The University of Chicago, Chicago IL, 60637, USA; 2 Howard Hughes Medical Institute, The University of Chicago, Chicago IL, 60637, USA

## Abstract

**Background:**

Counting *k*-mers (substrings of length *k *in DNA sequence data) is an essential component of many methods in bioinformatics, including for genome and transcriptome assembly, for metagenomic sequencing, and for error correction of sequence reads. Although simple in principle, counting *k*-mers in large modern sequence data sets can easily overwhelm the memory capacity of standard computers. In current data sets, a large fraction-often more than 50%-of the storage capacity may be spent on storing *k*-mers that contain sequencing errors and which are typically observed only a single time in the data. These singleton *k*-mers are uninformative for many algorithms without some kind of error correction.

**Results:**

We present a new method that identifies all the *k*-mers that occur more than once in a DNA sequence data set. Our method does this using a Bloom filter, a probabilistic data structure that stores all the observed *k*-mers implicitly in memory with greatly reduced memory requirements. We then make a second sweep through the data to provide exact counts of all nonunique *k*-mers. For example data sets, we report up to 50% savings in memory usage compared to current software, with modest costs in computational speed. This approach may reduce memory requirements for any algorithm that starts by counting *k*-mers in sequence data with errors.

**Conclusions:**

A reference implementation for this methodology, BFCounter, is written in C++ and is GPL licensed. It is available for free download at http://pritch.bsd.uchicago.edu/bfcounter.html

## Background

With recently-developed methods for massively parallel DNA sequencing it is now practical for individual labs to perform whole-genome or transcriptome sequencing of a wide variety of organisms, and to perform metagenomic sequencing of environmental samples. Additionally, these new sequencing technologies are becoming widely used for reduced representation sequencing and genotyping of non-model organisms [1,2], including those with no available genome sequence.

Each of these applications involves *de novo *assembly from very large numbers of short reads. Despite progress in recent years, *de novo *assembly remains a computationally challenging task. The current research for assembly with short reads is focused on *de Bruijn *graph methods [3-7]. The nodes in a *de Bruijn *graph are the *k*-mers of a pre-specified length *k *that are contained within the sequencing reads. Two *k*-mers are connected in the graph if they are adjacent in at least one sequencing read. Although *de Bruijn *graphs provide a nice conceptual framework that cuts down on computation time, the size of the graph can be very large, typically including billions of *k*-mers for vertebrate-sized genomes.

In order to deal with the computational challenges of working with such large data sets, a number of methods have been proposed for storing *k*-mers efficiently. Most *de Bruijn *graph assemblers store *k*-mers using 2 bits to encode each nucleotide, so that each *k*-mer  takes bytes. The *k*-mers are then stored in a hash table, usually with some associated information such as coverage and neighborhood information in the *de Bruijn *graph. The exact memory usage depends on the hash table used; for example, the assembly software ABySS [6] uses the Google *sparsehash *library, which has minimal memory overhead http://code.google.com/p/google-sparsehash/. Additionally, ABySS can share the memory load across multiple machines, splitting up the hash table so that each potential *k*-mer is assigned to a unique machine, although this setup has more communication overhead across machines and requires additional work by the end user. A recently-developed program named Jellyfish is specifically designed for *k*-mer counting (for *k*-mers of up to 32 bp) [8]. It uses a "quotienting" technique [9] to reduce the space needed to store each *k*-mer in a hash table, and it achieves much lower memory usage than other available methods. Additionally, [10] show how to compress both the *de Bruijn *graph and the *k*-mer coverage counts to nearly the optimal. However this compression is done after all the *k*-mers have been counted, in contrast to Jellyfish.

A complementary strategy for reducing memory usage is based on the observation that in current data sets, a large fraction of the observed *k*-mers may arise from sequencing errors. Most of these occur uniquely in the data, and hence they greatly increase the memory requirements of *de novo *assembly without adding much information. For this reason, it is frequently helpful to either discard unique *k*-mers prior to building the graph, or to attempt to correct them if they are similar to other, much more abundant, *k*-mers [11-14]. For example, the team that sequenced the giant panda genome obtained 56-fold coverage of the 2.4 GB genome on the Illumina sequencing platform [11]. Using a supercomputer with 512 GB of RAM, the authors counted a total of 8.62 billion 27-mers. After removing or correcting low-coverage *k*-mers, they eliminated 68% of the observed *k*-mers, reducing the total number to just 2.69 billion. Their genome assembly was based on this reduced set.

More generally, while the number of true *k*-mers in a genome sequence is at most the genome length, *G *(or less in practice, due to repeats), the number of spurious *k*-mers grows almost linearly with sequencing depth. To illustrate this, if we assume a uniform error rate *α *per nucleotide, then the expected number of spurious *k*-mers at sequence coverage *C *is , where *l *is the length of sequence reads. (This calculation ignores the rare events in which an identical sequencing error occurs more than once, and that error rates are typically highest near the ends of reads.) Then for example, at an error rate of 1% per base, read length of 100 bp, and *k *= 31, the number of spurious *k*-mers would exceed the genome length *G *at just 5.33-fold coverage.

However, even the seemingly simple goal of eliminating singleton, or low coverage, *k*-mers is computationally demanding in practice, since we do not know *a priori *which *k*-mers have low coverage. An obvious approach would be to simply load all observed *k*-mers into a hash table while counting the number of occurrences of each. But this task alone can easily overwhelm the memory of standard high performance machines.

The goal then is to implement a method for identifying unique *k*-mers (or more generally, *k*-mers that occur *< n *times), that makes highly efficient use of memory while providing efficient storage of *k*-mers with fast insertion and query times. The problem of counting the number of distinct *k*-mers is much easier if we are willing to settle for an approximate answer that works with high probability [15].

Here, we describe an approach to solving this problem by storing an implicit and highly compact representation of the observed *k*-mers, known as a Bloom filter. A reference implementation, implemented in a C++ program called BFCounter, is freely available. We show empirical results of applying this method to published sequencing data. We also discuss possible extensions and further applications of the method.

## Results and Discussion

### The Bloom Filter

The Bloom filter is a probabilistic data structure supporting dynamic set membership queries with false positives [16]. It allows us to identify in an extremely compact way all *k*-mers that are present more than once in a data set, while allowing a low rate of false positives. Bloom filters have been used widely in computing applications, but to date rarely in bioinformatics, but see [14,17,18].

**Figure 1:**
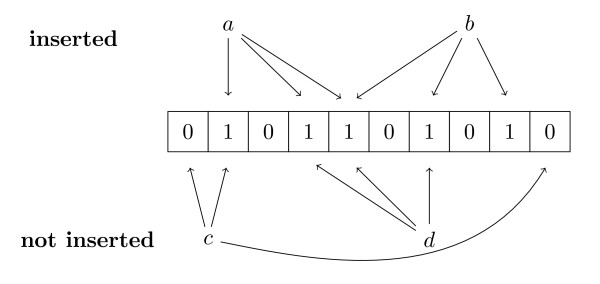
**Bloom filter example**. An example of a Bloom filter with three hash functions. The *k*-mers *a *and *b *have been inserted, but *c *and *d *have not. The three hash functions are represented with arrows, and the bits corresponding to the hashes for *a *and *b *have been set to 1. The Bloom filter indicates correctly that *k*-mer *c *has not been inserted since not all of its bits are set to 1. However, *k*-mer *d *is an example of a false positive: it has not been inserted, but since its bits were set to 1 by the insertion of *a *and *b*, the Bloom filter falsely reports that *d *has been seen already.

The essential idea is illustrated in Figure 1. The Bloom filter is a bit array *B*, initialized to be 0 at every position. We also define a set of *d *hash functions, *h*_1_, ..., *h_d_*, where each hash function maps a given *k*-mer *x *to a location in *B*.

In order to insert a *k*-mer *x *into the Bloom filter, we set all of the *d *corresponding locations in *B *to be 1; that is, we set *B*[*h_i_*(*x*)] = 1 for *i *= 1, ..., *d*. Then, to determine whether a *k*-mer *y *has been inserted, we simply check whether each of the corresponding hash positions is 1: i.e., whether *B*[*h_i_*(*y*)] are all set to 1 for *i *= 1, ..., *d*. If this is the case, then we infer that *y *has probably been seen before. By construction, this procedure correctly identifies every *k*-mer that is present more than once in the data; however, the cost of very efficient memory usage is that we accept a low rate of false positives in which we infer that *y *has been seen previously, but in fact it has not.

The Bloom filter has a tradeoff between memory usage (i.e., the number of bits used) and the false positive rate. When storing *n k*-mers in a Bloom filter of *m *bits, and using *d *hash functions, the false positive rate is approximately . Given *n *and *m*, the optimal number of hash functions that minimizes the false positive ratio is [19]. In practice we may have a rough idea in advance about *n*, the number of *k*-mers, and we can select *m *as a fixed multiple of *n*. For example using *m *= 8 · *n *(which corresponds to storing one byte per *k*-mer), and *d *= 5 gives a false positive ratio of 2.16%. Many variations and improvements have been proposed for Bloom filters [20,21]; or see [19] for a survey.

### Storing and counting *k*-mers using the Bloom Filter

To count all non-unique *k*-mers we use a Bloom filter *B *and a simple hash table *T *to store *k*-mers. The Bloom filter keeps track of *k*-mers we have encountered so far and acts as a "staging area", while the hash table stores all the *k*-mers seen at least twice so far. The idea is to use the memory-efficient Bloom filter to store implicitly all *k*-mers seen so far, while only inserting non-unique *k*-mers into the hash table.

Initially both the Bloom filter and the hash table are empty. All *k*-mers are generated sequentially from the sequencing reads. Note that in most applications we do not need to distinguish between a *k*-mer and its reverse complement sequence. Thus, as we read in each *k*-mer we also consider the reverse complement of that *k*-mer and then work with whichever of the two versions is lexicographically smaller (we refer to the smaller sequence as the "canonical *k*-mer").

For each *k*-mer, *x*, we check if *x *is in the Bloom filter *B*. If it is not in *B *then we update the appropriate bits in *B *to indicate that it has now been observed. If *x *is in *B*, then we check if it is in *T*, and if not, we add it to *T*.

**Figure 2:**
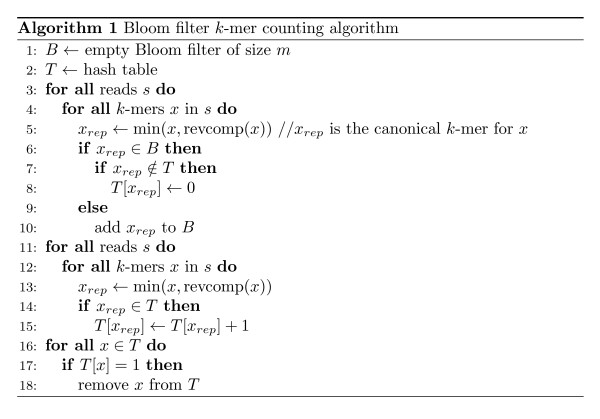
**Algorithm pseudocode**. A pseudocode for the Bloom filter *k*-mer counting algorithm.

This scheme guarantees that all *k*-mers with a coverage of 2 or more are inserted into *T*. However a small proportion of unique *k*-mers will be inserted into *T *due to false positive queries to *B*. After the first pass through the sequence data, one can re-iterate over the sequence data to obtain exact counts of the *k*-mers in *T *and then simply delete all unique *k*-mers. The time spent on the second round is at most 50% of the total time, and tends to be less since hash table lookups are generally faster than insertions. A detailed pseudocode is given in Figure 2.

It is also possible to obtain approximate *k*-mer counts by iterating only once over the sequence reads. In this case we record a coverage count of 2 when first inserting a *k*-mer into the hash table *T*, and subsequently increment the counter for each additional observation of this *k*-mer. This means that the coverage counts for some *k*-mers are 1 higher than the true value, and some *k*-mers in *T *are in fact false positives (i.e., present only once).

### Higher Coverage Cutoffs

For some applications a higher coverage cutoff may be required to either filter out sequencing errors or to simply extract sequences of interest. The algorithm can be extended to use counting Bloom filters, where each bit in the bit array is now replaced with a counter that uses only a small number of bits. If the desired minimum coverage is *c *we use an array of *m *⌈log_2_(*c*)⊥-bit counters. The counting Bloom filter was introduced by [20] to allow for deletions, but here we use the counts directly.

To check if a *k*-mer should be inserted into the hash table *T *we look to see if all of *B*[*h_i_*(*x*)] are equal to *c *- 1. Otherwise we insert it into the Bloom filter. When inserting a *k*-mer *x*, we set

for *i *= 1, ..., *d*. Note that for a *k*-mer *x*, min{*B*[*h_i_*(*x*)]|*i *= 1, ..., *d*} gives an upper bound on the number of occurrences of *x *so far. Of course the basic version simply corresponds to the case of *c *= 2.

### Parallelizability

The algorithm is presented above as a standard single processor program and our current implementation is not multi-threaded.

Nonetheless it would be possible to speed up the operations using multiple cores with lock-free data structures. This would require a non-blocking implementation of the hash table [22] and a modification to the Bloom filter. The bit array in the Bloom filter is implemented as an array of word-sized integers, usually 32 or 64 bits. To avoid accidental collisions where two bit locations in the same word are updated, one can use "compare-and-swap" (CAS) operations on words to ensure atomic updates of each bit independently.

Since the role of the Bloom filter is to keep track of *k*-mers seen previously, this scheme could plausibly fail in the unlikely event that two occurrences of the same k-mer are inserted into the Bloom filter simultaneously by different threads. In this case the two threads would both query the Bloom filter for a *k*-mer, *x*, and after both receive a negative answer the two threads would insert *x *simultaneously. If *x *occurs exactly twice in the data set then we would fail to record it in the hash table and get a false negative, although this type of false negative seems unlikely to be a serious concern in practice. However this can be fixed by extending the Bloom filter data structure to return the number of bits set to 1 when querying, and the number of bits changed from 0 to 1 when inserting. This makes insertion atomic, each thread can then determine when inserting a new *k*-mer into the Bloom filter whether any other threads were inserting the same *k*-mer simultaneously by comparing the number of bits changed from 0 to 1. If the two numbers do not match, we can infer that some other thread had already inserted the *k*-mer into the Bloom filter and proceed with inserting the *k*-mer into the hash table.

### Implementation

We implemented this algorithm in a program called BFCounter in C++, available from http://pritch.bsd.uchicago.edu/bfcounter.html The source code is licenced under a GPL licence. For the implementation we used the Google *sparsehash *library and a Bloom filter library by A. Partow http://www.partow.net/programming/hashfunctions/index.html. We store a 1-byte counter for each *k*-mer and by default *k*-mers take 8-bytes of memory with a maximum *k *of 31, although if desired, larger *k*-mers can be specified at compile time. We require the user to specify an estimate for the number of *k*-mers in the sequencing data and use a Bloom filter with 4 times as many bits as the expected number of *k*-mers this corresponds to a memory usage of 4-bits per *k*-mer and the optimal number of hash functions functions for the Bloom filter is *d *= 3.

### Example data sets

To illustrate the performance of the new method, we describe the analysis of two data sets of sequencing reads from human genomic DNA. The first data set consists of 7.5 M 100 bp paired-end reads from the Illumina platform that mapped to Chromosome 21. These data, from HapMap individual NA19240, are available from Illumina at http://www.illumina.com/truseq/tru_resources/datasets.ilmn. This data set corresponds to approximately 32-fold coverage of Chromosome 21, a coverage-level that is typical of many contemporary sequencing studies. Since the reads have already been mapped to a genome this likely represents a cleaner data set (i.e., with fewer errors and lower repeat content) than we would expect to get from unprocessed sequence data.

The second data set consists of genome-wide sequence data from the 1000 Genomes Project Pilot II study [23]. Individual NA19240 was sequenced at 40-fold coverage, using 2.66 billion 36 bp paired-end Illumina reads. The data were filtered to remove sequences with low quality scores and missing basecalls; they are available at ftp://ftp.1000genomes.ebi.ac.uk/vol1/ftp/data/NA19240/sequence_read/.

**Figure 3:**
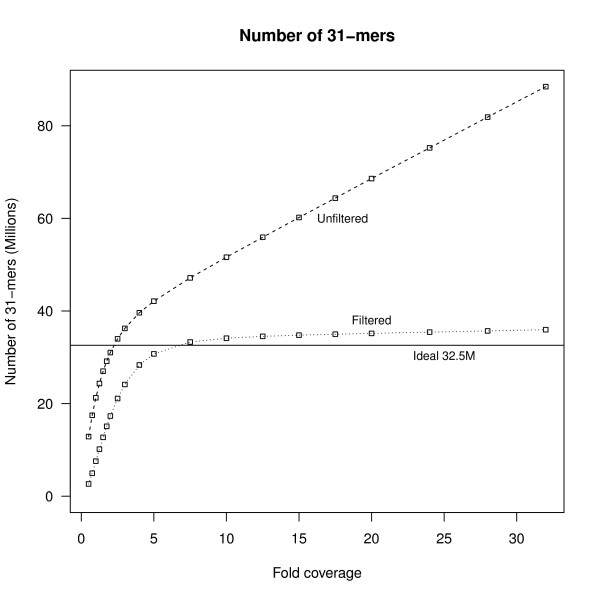
**Number of *k*-mers**. The plot shows the number of distinct *k*-mers found in the sequencing data from chr21 at different coverage levels, based on random subsampling of the data. The total number of distinct *k*-mers in the hg18 genome sequence of chr21 is 32.5 million *k*-mers. Unfiltered, the number of *k*-mers found increases at a steady rate after 5-fold coverage. When unique *k*-mers are removed, the number of filtered *k*-mers approaches the ideal number at around 7-fold coverage and the rate of increase is significantly reduced.

Our first application is to the 32-fold sequence data from Chromosome 21 data. We collected all *k*-mers from the sequencing reads, using *k *= 31. Figure 3 shows the distribution of the number of times each *k*-mer is seen in the input data. Out of 80.4M observed *k*-mers, slightly more than half (48.7M) are observed only a single time. The vast majority of these singleton *k*-mers (99.87%) are not found in the reference genome and hence are most likely due to sequencing errors, thus supporting the approach of discarding or correcting these unique *k*-mers.

**Figure 4:**
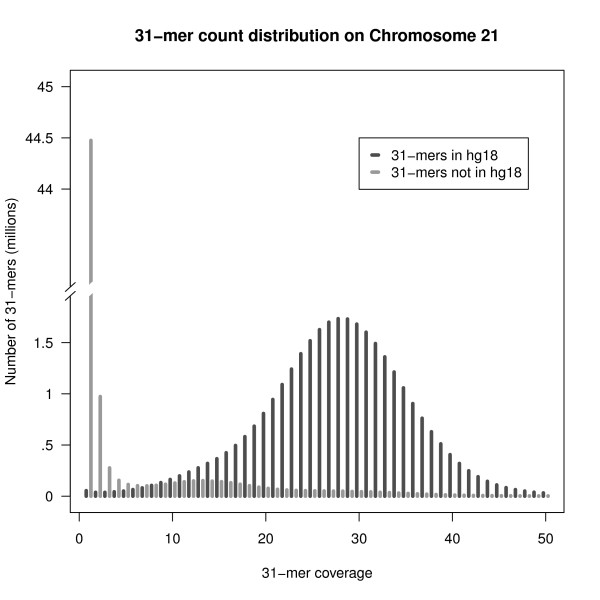
***k*-mer distribution**. Distribution of coverage levels for *k*-mers in the sequence reads from chromosome 21. There is a clear distinction between the coverage levels of the 31.7M observed *k*-mers that are found in the hg18 reference genome sequence compared to the 48.7M *k*-mers that are not in hg18. Of the *k*-mers not found in hg18, 44.5M or 99.87%, are observed only once, and are likely sequencing errors. A small fraction of *k*-mers that do not match hg18 are observed many times in the data; these likely represent SNP differences between the sequenced individual and hg18 and would be retained by the Bloom filter.

Figure 4 illustrates how the total number of *k*-mers, and the number of nonunique *k*-mers increases with sequencing depth for this data set. For the unfiltered *k*-mers we see the same behavior with increasing coverage as expected from the Introduction: namely, the total number of *k*-mers found increases approximately linearly for coverage levels greater than about 5X. This increase is almost completely due to the increase in unique *k*-mers that contain errors. In contrast, the number of non-unique *k*-mers is only slightly more than the expected number based on the number of distinct *k*-mers in the hg18 genome sequence from Chromosome 21.

To evaluate the computational performance of BFCounter we compared it to Jellyfish [8] and to a naive *k*-mer counting program without any filtering. All comparisons were done on a 64-bit x86 Intel Xeon machine with 8 cores at 2.4 GHz and 144 GB of memory running Linux kernel version 2.6.18. The disks were all from shared network through Lustre. All time measurements were done with the time unix command and memory usage was measured using strace.

The naive version simply stores all *k*-mers explicitly in a Google *sparsehash *hash table and skips the filtering step. Jellyfish is a sophisticated *k*-mer counting program that features support for multicore machines. Furthermore Jellyfish stores an implicit representation of *k*-mers in a hash table to save memory. The authors of the Jellyfish program recently showed that their method provides large memory savings compared to other traditional methods for *k*-mer counting. Jellyfish requires us to prespecify the size of the hash table to use; if the hash table fills up, the results are written to disk and merged later. To compare the programs we found the minimum size so that Jellyfish could keep all *k*-mers in memory. For the second data set Jellyfish could not fit all *k*-mers in memory with default parameters. To fit the hash table in memory we needed to set the number of reprobes to 255 by running Jellyfish with the -p 255 option. For timing comparisons we run Jellyfish in serial mode.

**Figure 5:**
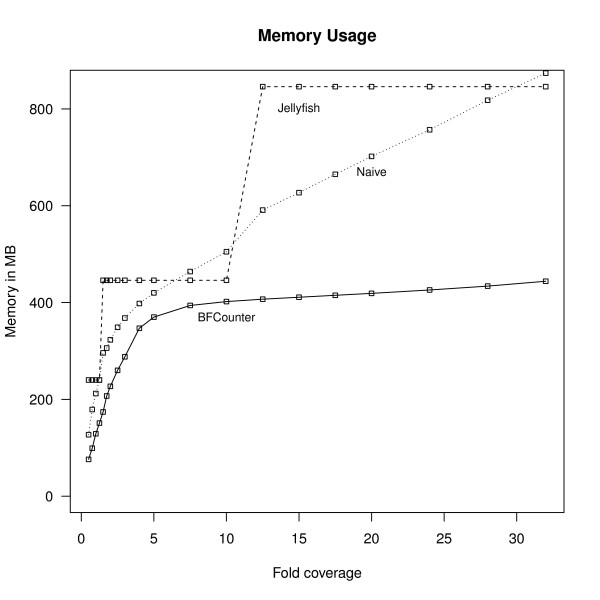
**Memory usage**. The memory usage of the three programs at different coverage levels (Chromosome 21 data). Note that Jellyfish and the naive counter are storing *all k*-mers while BFCounter filters out most unique *k*-mers without storing them explicitly in memory. The memory usage of BFCounter and the naive version roughly mimic the shape for the number of filtered *k*-mers in Figure 3. The discrete jumps in the memory usage of Jellyfish are due to implementation details as the size of the hash table has to be a power of 2.

The increase in the number of *k*-mers affects the memory consumption directly. Figure 5 plots the memory requirements of BFCounter, Jellyfish and the naive version. The increase in memory levels off for BFCounter after about 7-fold coverage, whereas for the naive version and Jellyfish the memory increases steadily as the number of *k*-mers grows.

Table 1 presents the memory and time requirements for the three methods when applied to the second data set (40-fold coverage of a human genome with 36 bp reads). For this analysis we set the *k*-mer length *k *= 25, which strikes a balance between the number of *k*-mers produced by each read, here 11, and the specificity of the *k*-mers. Although for this data set the average basepair coverage is fixed, the *k*-mer coverage decreases with *k*. On the other hand increasing *k *gives more observed *k*-mers, since sequencing errors can generate up to *k *unique *k*-mers.

**Table 1:** Memory usage and Time for whole genome data

Program	Time (hrs)	Memory (GB)
BFCounter	23.82	42
Jellyfish	8.03	71
Naive*	>26.38	>128

Memory and time usage for the 1000 Genomes data set (40-fold coverage of individual NA19240). *The naive version ran out of memory after processing 84.7% of the reads.

There are 12.18 billion *k*-mers present in the sequencing reads, of which 9.35 billion are unique and 2.83 billion have coverage of two or greater (compared to 2.37 billion distinct 25-mers in the hg18 genome sequence). When BFCounter was run, about 0.5 billion of the unique *k*-mers were stored in the hash table after the first phase which corresponds to a 5.3% false positive rate for the Bloom filter. Thus, BFCounter stored 27% of the original *k*-mers after the first pass, and this was cut to 23% after false positives were removed.

As may be seen from the table, BFCounter uses considerably less memory than either Jellyfish or the naive hash table method. Indeed the naive method ran out of memory and was unable to complete. However, BFCounter takes approximately three times longer to run as Jellyfish. Part of the difference in speed is due to BFCounter taking a second pass through the data to obtain exact *k*-mer counts (which may not be essential for all applications).

## Conclusions

Counting *k*-mers from sequencing data is an essential component of many recent methods for genome assembly from short read sequence data. However, in current data sets, it is frequently the case that more than half of the reads contain errors and are observed just a single time. Since these error-containing *k*-mers are so numerous, they can overwhelm the memory capacity of available high-performance machines, and they increase the computational complexity of downstream analysis.

In this paper, we describe a straightforward application of the Bloom filter data structure to help identify and store the reads that are present more than once (or more than *n *times) in a data set, and are therefore far more likely to be correct. By doing so, we achieve greatly reduced memory requirements compared to a naive but memory-efficient hash table method, as well as to Jellyfish (which has been highly optimized for memory efficiency, while storing all *k*-mers). For many applications, it may be sufficient to simply ignore the unique *k*-mers (as was done for the panda genome); alternatively, users may prefer to "correct" reads by comparing unique *k*-mers to common *k*-mers [11-14]. In summary, the approach presented here could be straightforwardly incorporated into a wide variety of algorithms that start by counting *k*-mers.

Our method trades off reduced memory usage for an increase in processing time. In many cases the memory limitation is a hard threshold and the counting of *k*-mers is only run once and a fixed set of *k*-mers is stored for future computation. For genome assembly methods the construction of de Bruijn graphs dominates memory consumption [7] and the time for completion can be several days [13], depending on the amount of postprocessing.

## Authors' contributions

PM and JKP contributed ideas and participated in writing this article. PM designed the algorithm, implemented the software and ran the experiments. Both authors read and approved the final manuscript.

## Funding

This work was funded by a grant from the National Institutes of Health: MH084703. JKP is supported by the Howard Hughes Medical Institute.
